# Entropy-Based Metrics for Occupancy Detection Using Energy Demand

**DOI:** 10.3390/e22070731

**Published:** 2020-06-30

**Authors:** Denis Hock, Martin Kappes, Bogdan Ghita

**Affiliations:** 1 Faculty of Computer Science and Engineering, University of Applied Sciences Frankfurt am Main, 60318 Frankfurt am Main, Germany; kappes@fb2.fra-uas.de; 2 School of Engineering, Computing and Mathematics, Plymouth University, Plymouth PL4 8AA, UK; bogdan.ghita@plymouth.ac.uk

**Keywords:** energy demand, entropy applications, privacy

## Abstract

Smart Meters provide detailed energy consumption data and rich contextual information that can be utilized to assist electricity providers and consumers in understanding and managing energy use. The detection of human activity in residential households is a valuable extension for applications, such as home automation, demand side management, or non-intrusive load monitoring, but it usually requires the installation of dedicated sensors. In this paper, we propose and evaluate two new metrics, namely the sliding window entropy and the interval entropy, inspired by Shannon’s entropy in order to obtain information regarding human activity from smart meter readings. We emphasise on the application of the entropy and analyse the effect of input parameters, in order to lay the foundation for future work. We compare our method to other methods, including the Page–Hinkley test and geometric moving average, which have been used for occupancy detection on the same dataset by other authors. Our experimental results, using the power measurements of the publicly available ECO dataset, indicate that the accuracy and area under the curve of our method can keep up with other well-known statistical methods, stressing the practical relevance of our approach.

## 1. Introduction

The smart grid is generally seen as the next-generation power system, connecting all of the actors of the energy system and optimizing the reliability and efficiency of electricity supply and consumption. The advent of ubiquitous IP-based data exchange in modern power grids and the availability of residential energy measurements in low-voltage networks boosted the interest towards appropriate data assessment for time-dependent market prices, non-intrusive load monitoring, or demand side management.

Many of these methods ultimately aim to save energy by optimizing the customers consumption behaviour. Other authors [1,2] provided evidence that buildings are responsible for a major part of the global energy consumption. According to Vafeiadis et al. [3], the detection of occupancy provides the ability to apply several automation applications that may contribute to the reduction of energy consumption. Candanedo et al. [4] found that an accurate determination of occupancy in buildings can save energy, up to 40%.

In this paper, we propose an effective, entropy-inspired profiling mechanism that consolidates the temporal distribution of energy consumption in order to implement an indicator for occupancy. The occupancy is an interesting metric for many new applications in the smart grid, e.g., monitoring and forecast, dynamic prices, or typical load classification. Detecting when a residential household is occupied, is still a challenging process, which is typically solved by the intrusive deployment of dedicated sensors, such as infrared sensors, magnetic switches, or cameras. Other authors [5,6] generally agree that these setups require the expensive deployment, calibration, and maintenance of hardware. Furthermore, a wide range of machine learning algorithms, which can indicate occupancy using the energy demand, are unsuitable due to the requirement of historic data and high resolution data. Our contribution is that we reveal this information without a priori information, which allows its use without expensive and laborious training. Furthermore, our algorithm can also cope with low resolution measurements.

As human activities that take place without appliances are not reflected in energy demand, we define the term occupancy in this paper as an interaction with electrical appliances or activity of devices. Obviously, mispredictions of occupancy in this sense are sometimes possible as, e.g., a cleverly randomized use of time-switches cannot be distinguished from a human user doing the same activity. While approaches that are aimed at detecting or predicting actual human presence in homes might appear more powerful, we argue that our proposed approach is more efficient as measuring, modelling, and predicting actual human activities, which is either intrusive or difficult. The differences between these approaches are, in fact, negligible from a practical point of view when considering the use of such data for non-intrusive load monitoring, anomaly detection, and energy forecasts.

The idea to use the entropy to analyse the regularity of energy demand is intuitive, but it contains many hidden challenges. We showcase methods to use the energy demand, horizontally with a sliding window and vertically with intervals, as input data for the entropy. We analyse the respective parameters, such as the window size and number of intervals, and interpret the meaning of a high and low entropy. Our aim is to create a comprehensible metric that can be used to identify or distinguish the behaviour of residential households better than raw data.

In the remainder of this paper, we first present an overview of the statistical entropy and discuss details of two independent metrics. We highlight their advantages and limitations regarding their conclusiveness with respect to residential energy demand. Next, we evaluate our metrics by presenting several practical experiments, which illustrate their functionality and features. Our experimental results, which were obtained by applying entropy to real world smart meter data of the public available Electricity Consumption & Occupancy (ECO) dataset, show that the proposed metric can indeed detect occupancy. A comparison of our results with other well-known statistical methods, followed by a summary and potential future work avenues, concludes this paper.

## 2. Related Work

Many authors agree that residential energy demand can provide unique insights into household characteristics or individual behaviours. An energy provider can use these information to better monitor the complex consumption behaviour, improve the prediction of certain consumer profiles, and suggest optimal energy pricing for particular groups. In particular, Anderson et al. [7] discussed and examined a great many of literature that suggests that we can use residential energy demand to indicate household characteristics and associate consumption patterns to particular consumer groups.

Previous studies of Molina et al. [8] used density-based clustering and supervised learning to identify private information about consumers. Research conducted by Carroll et al. [9] and Mcloughlin et al. [10] suggests that there is a relationship between load curves and the employment status as well as the presence of children. Kolter et al. [11] analysed the relation between demand and building properties, such as the number of rooms and the building value. Beckel et al. [12] extracted the number of occupants from energy demand. Newing et al. [13] associated energy consumption patterns with particular dwellings, income, and number of children.

Nguyen and Aiello [14] as well as Labeodan et al. [15] provide a comprehensive list of approaches to capture occupancy, which are mostly based on sensor data. Kleiminger et al. [5], who provided the Electricity Consumption & Occupancy (ECO) dataset, implemented a method that was based on supervised machine learning algorithms to detect human presence. Jin et al. [6] performed occupancy detection on both residential and commercial buildings. Becker et al. [16] computed the occupancy with unsupervised occupancy detection algorithms on three public datasets. Peng et al. [17] used unsupervised and supervised learning machine learning methods to predict the occupancy of office buildings.

The major contributions of this paper include the application of an entropy-inspired metric to profile energy demand. The entropy has been extensively applied in other areas such as health care [18], biodiversity assessment [19], or network monitoring [20].

Some of the authors applied the entropy to topics related to the smart grid, but not occupancy detection. E.g., Iranmanesh et al. [21] used the entropy based mutual information method in order to select relevant input features for energy forecasting. Ruiz et al. [22] used the entropy to measure the volatility of energy price markets and relate them to historical events or climatic factors. Gao et al. [23] used the entropy to assess risks of the electricity market. Hock et al. [24] used the entropy to detect energy theft by comparing multiple households.

In contrast to most conventional methods, our metric does not require large amounts of historical data to build a statistical model and it is relatively simple to compute. To the best of our knowledge, an entropy-inspired metric has not been proposed to analyse energy demand before.

## 3. Method

In the following, we will briefly outline the rationale behind our approach to use a somewhat complex metric to reliably detect events, such as changes in demand and energy usage pattern. Our objective is to detect electric events pointing to occupancy. Due to appliances with high demand, typical statistical ratios are insufficient for reflecting occupancy, because the amplitude of energy intensive or energy saving appliances do not necessarily translate into different human activities. Hence, we propose using regularity or randomness, in other words temporal changes of demand, as a benchmark for occupancy.

Energy demand is not random—the patterns of human activity, given by the stacked load shapes of individual appliances, are often gathered in a few periods per day and repeat daily according to the consumption habits of the household. We aim to observe these sections, which appear due to a high variability as outlier, through measuring entropy.

The entropy is a convenient way to detect outliers in the regularity of energy demand. The distribution of energy demand can be measured horizontally or vertically. By analysing the distribution of demand over time (x-axis), we may find out that most of the demand appears at a specific time, which is an outlier. By analysing the distribution of measurements (y-axis), we may find out that there are only few measurements with high power, which also is an outlier.

Although entropy originated in thermodynamics, Shannon argued with his application of the entropy to information theoretic problems—as a quantitative and qualitative technique for understanding ’uncertainty’—that entropy has a deeper meaning. It is well known in coding theory that the entropy of a discrete random variable quantifies the average length of the encoding of the random variable. Moreover, Shannon’s entropy measures the average uncertainty, also referred to as information content. The uncertainty is maximized when the outcomes of the random variable are equally likely, which corresponds to a uniform distribution. As will be shown later, entropy-based measures capturing the entropy of energy-consumption over time are useful in detecting occupancy.

Formally, let $L=\{a_{1},a_{2},\ldots ,a_{n}\}$ be a dataset where *n* is the cardinality of *L*. Moreover, let $x_{1},x_{2},\ldots ,x_{n}$ denote the frequency of each element in *L* in some sequence *X* of elements from the set and $m=\sum_{i=1}^{n}x_{i}$ implies the number of all observations. Subsequently, the entropy H(X) is defined by

(1) $$\begin{array}{cc} H(X) & =-\sum_{i=1}^{n}\frac{x_{i}}{m}\cdot \log_{2}\left(\frac{x_{i}}{m}\right) \end{array}$$

Please note that $0\leq H\left(X\right)\leq \log_{2}\left(n\right)$ where H(X)=0 is assumed if only one element of *L* occurs in *X* (by convention, $0\cdot \log_{2}\left(0\right)=0$) and $H\left(X\right)=\log_{2}\left(n\right)$ if all elements of *L* occur in *X* with the same frequency. As $\frac{x_{i}}{m}$ is synonymous for the occurrence probability, we can extend the interpretation to the statistical distribution of the underlying data: an entropy that is close to $\log_{2}\left(n\right)$ reads as ‘random’, because the elements appear almost equally often, whereas an entropy that is close to zero reads as ‘skewed’, because few elements appear more frequent.

As we use the entropy not in a traditional sense, but as a metric to mathematically trace outliers in a distribution of measurements, the practical function as a metric, which encodes distributions without losing information on outliers, is the most important aspect for us.

**Figure 1:**
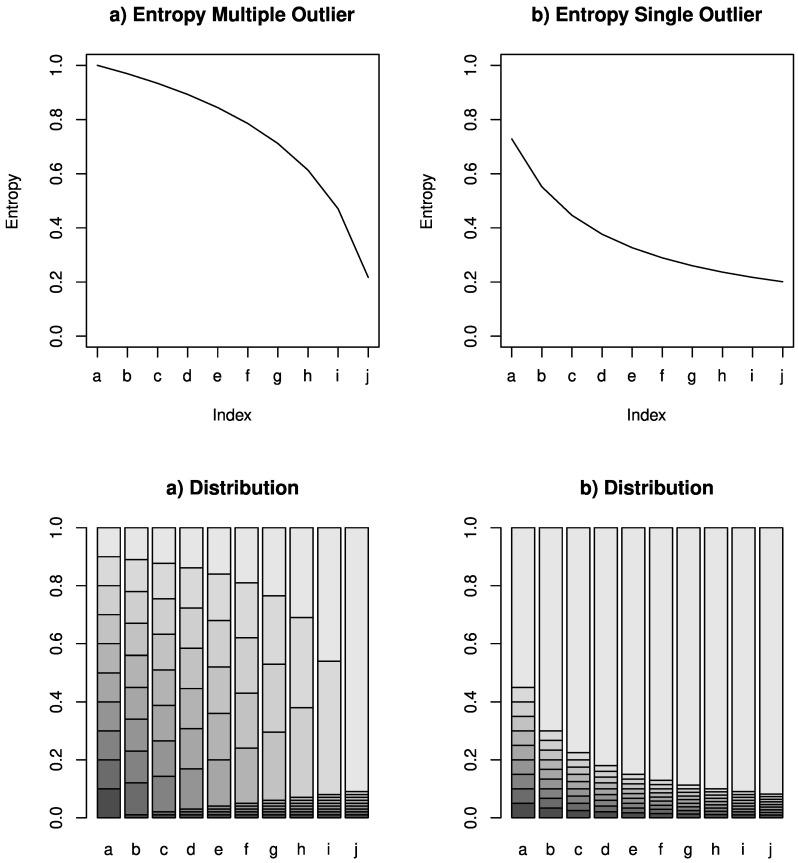
Entropy for uniform and heavily skewed distributions.

Figure 1 illustrates the entropy (top) for a corresponding distribution (bottom) with a generated example. The two figures at the bottom show bar plots with 10 different bars, called (a–j), each visualising a distributions. The distributions are from left to right more and more skewed to showcase the resulting entropy, which decreases with the skewness. The left-hand side focuses on multiple outliers, while the right-hand side focuses on single outliers.

Apart from the entropy-inspired metric, other distance functions may also work in order to detect outlier in the distribution. Our metrics result looks strongly correlated to the standard deviation (σ). However, the σ of a vector increases with the distance between values, while the entropy only considers the number of unique values. E.g., for a vector $v_{1}=\left(1\;W,1\;W,1000\;W,1000\;W\right)$ and $v_{2}=\left(1\;W,250\;W,750\;W,1000\;W\right)$. The σ for $v_{1}$ is higher than for $v_{2}$. However, the entropy for $v_{2}$ is higher than for $v_{1}$.

## 4. Application

Subsequently, we illustrate metrics that are based on Shannon’s entropy, as a unified analytic approach to discover changes of energy demand over time. As previously explained, the range of the entropy is profoundly affected by the size of the dataset. In our case, it coincides with the finite amount of energy demands *n*, which, in turn influence its range [0, $\log_{2}\left(n\right)$]. Hence, we will discretise continuous sets, such as R, in order to use them as input parameter for our entropy-measure.

Following the reasoning of the previous chapter, we introduce two independent methods to observe the uniformity of energy demand vertically and horizontally resulting in two opposite interpretations, different from the information theoretic interpretation, aiming to significantly mirror the changes in energy demand pattern. While our first method sliding window entropy employs the probability to see demand during a certain time, the second method interval entropy conducts the probability to see demand in a certain range. We aim to explain the different interpretation of the result, in contrast to the traditional Shannon entropy, and choose the best approach to detect occupancy.

Human activity stands out due to energy demand peaks. The sliding window entropy can detect such a peak if a single time window contains most of the total demand. Alternatively, human activity stands out due to high variance. The interval entropy can detect this variance if the power measurements are equally distributed over the measurement range.

### 4.1. Sliding Window Entropy

To summarize the sliding window entropy, we utilize the proportion of energy demand in a time window as input for the entropy to obtain a metric that explains the distribution of energy demand over one day. In contrast to the information theoretic entropy, our metric is maximized if the demand in all of the time windows is equal and the consumption is distributed uniformly throughout the day and minimized when the consumption is concentrated in a single interval. Hence, we assume occupancy when the entropy is minimized.

Consider a finite time series $T=a_{1},a_{2}\;\ldots \;a_{n},\;a_{i}\in R_{0}^{+}\;\forall \;1\leq i\leq n$, with *n* elements - representing energy demand. Subsequently, a sliding window X with size *m*, where the sliding step (1≤Δ≤m) defines the number of elements by which the window slides each iteration *t*, can be defined as $X_{t}=a_{t\Delta },a_{t\Delta +1},\ldots ,a_{t\Delta +m-1}$, which results in a total of $0\leq t\leq \frac{n-m}{\Delta }+1$ windows to cover all elements. The traditional entropy considers the occurrences $x_{i}$ over the data length *n*, which is equivalent to the probability to see a certain element $\frac{x_{i}}{n}$. In our energy usage context, the entropy considers the demand probability in a sliding window $P(X_{t})$, which is equivalent to the normalised integrated area under the demand line. We simplify the approximation of area, by dividing the demand of a window $h(X_{t})$, which is the sum of all measurements in a window, by the total demand h(T):

(2) $$\begin{array}{cc} P(X_{t}) & =\frac{h(X_{t})}{h(T)} \end{array}$$

We could also interpret the probability as the proportion of daily energy used during that sliding window. Note that the sum of all probabilities can be greater than 1, if the sliding windows overlap (Δ<m), which results in a scaled entropy.

**Figure 2:**
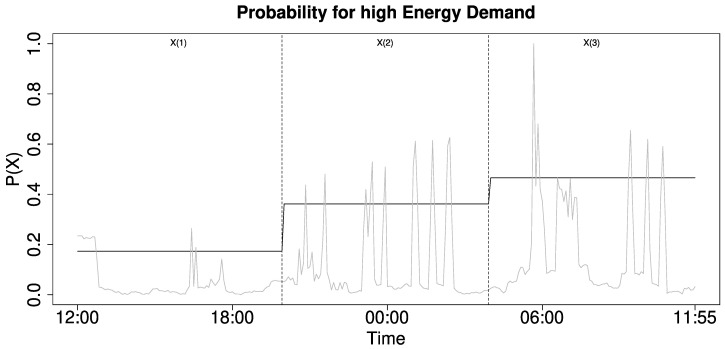
Integrated demand for three time windows.

Figure 2 illustrates the energy demand (grey line) divided into three time window X(1:3), m=8h, Δ=m (dotted lines), with the probability for energy demand delineated in black.

The entropy is maximized if the demand is distributed uniformly and minimized if the total demand is only present in a single sliding window. The result ranges from $\left[0,\log\left(\frac{n-m}{\Delta }+1\right)\right]$. Equation (3) illustrates how to calculate the entropy while using the above probability.

(3) $$\begin{array}{cc} H(X) & =-\sum_{t=0}^{\frac{n-m}{\Delta }+1}P\left(X_{t}\right)\cdot \log\left(P\left(X_{t}\right)\right) \end{array}$$

**Figure 3:**
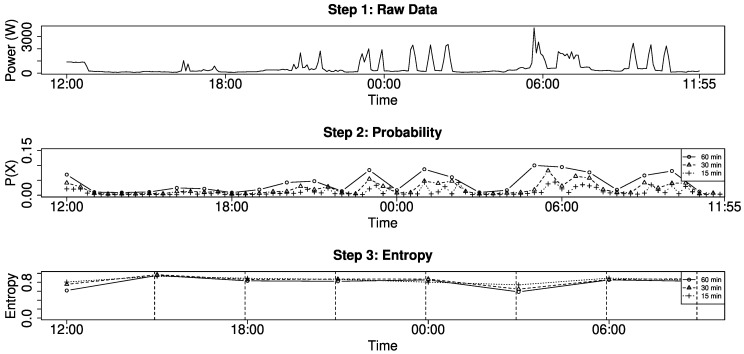
Probability to use energy in a certain time window.

Occupancy stands out due to the imbalance and change of energy consumption, which manifests here in form of demand peaks. High demand concentrated in a single time window results in a minimized sliding window entropy. Figure 3 visualizes the complete process to derive the occupancy from raw energy demand. We first compute the probability for high demand (middle) in three different resolutions and then compute the entropy for a time window of three hours using the probability as input data. In the first step, we define three different sliding windows X with $m_{1}$ = 15, $m_{2}$ = 30, and $m_{3}$ = 60 each with Δ=m over the total of n=1440 measurements. Step 2 shows the resulting 24 probabilities for $m_{1}$ (dot), 48 probabilities for $m_{2}$ (triangle) and 96 probabilities for $m_{3}$ (cross). Here, the sum of measurements in each sliding window is divided by the sum of the complete time series, in order to generate one probability value for each sliding window. In the last step, the probabilities derived from the previous time windows $P(X_{t})$ are used as input value to compute an entropy. In our example, we use three probabilities (dot), six probabilities (triangle), and 12 probabilities (cross), which always corresponds to three hours, as input to compute the entropy. This results in eight entropy values per day, but the amount of probabilities used to compute the entropy is of course a variable parameter.

In the resulting plot, we can clearly see the influence of several parameters, namely the number of time windows used as input for the entropy (middle) and the size of the time window for each entropy value (bottom). The number of probabilities changes the level of our output—the resulting entropy is smaller the more input values we have, while the pattern of our curve is unchanged. The entropy results in a filter operation over the energy demand and smaller time windows for the entropy would show more detail. There is a visible drop of the entropy whenever the amplitude significantly changes within a time window. For our decision, whether a time window represents occupancy or not, we can simply utilize a threshold. What may look unusual is that the difference between time windows is not considered in this metric, because the sum of probabilities of any time window result in 100%. Hence, two time windows with (0.1 W,1 W) and (1 W,100 W) result in the same entropy.

### 4.2. Interval Entropy

To summarize the interval entropy, we count the amount of measurements in each interval to compute a probability that results in a metric affected by the distribution between intervals. This metric is maximized if the demand values are uniformly distributed over all intervals, which is the direct opposite to our previously introduced sliding window entropy.

Consider a finite time series $T=a_{1},a_{2}\;\ldots \;a_{n},\;a_{i}\in R_{0}^{+}\;\forall \;1\leq i\leq n$, with *n* elements. Subsequently, a finite number *m* of equal-sized discrete intervals I covering a total range of r=max(T)−min(T), is given by $I_{i}=\left[\frac{r}{m}\cdot \left(i-1\right)+min\left(T\right),\frac{r}{m}\cdot i+min\left(T\right)\right]$, with 1≤i≤m.

Our entropy in Equation (4) represents the probability that the demand is an element from a certain interval I, where *n* is the number of elements in T and *m* is the number of intervals.

(4) $$\begin{array}{cc} Freq(I_{i}) & =\sum_{j=0}^{n}a_{j}\in I_{i}\\ P(I_{i}) & =\frac{Freq(I_{i})}{n} \end{array}$$

Equation (4) shows the computation of the input for the entropy. For each interval $I_{i}$, we count the number of measurements *a* using the function Freq() and then divide by the total number of measurements to get the probability. The entropy is maximized if the demand distributes an equal number of elements to each interval *m* and minimized if the demand involves only one interval. The result ranges between [0, log(m)]. Equation (5) show how to utilize the probability of each interval to compute the entropy.

(5) $$\begin{array}{cc} H(I) & =-\sum_{i=0}^{m}P\left(I_{i}\right)\cdot \log\left(P\left(I_{i}\right)\right) \end{array}$$

**Figure 4:**
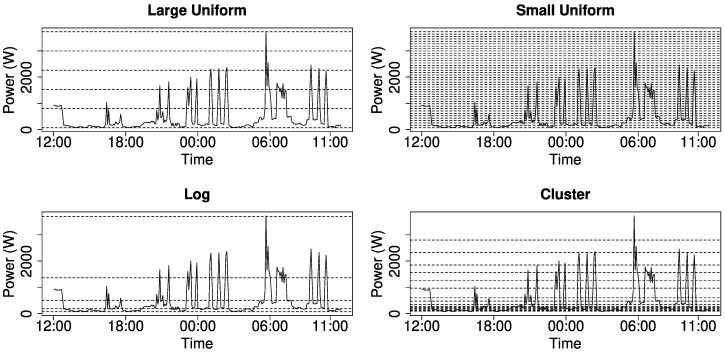
Energy demand in different intervals.

We experimented with different methods to generate intervals (see Figure 4)—the top figures show equally distributed intervals, while the bottom figures have intervals with dynamic size—namely, logarithmically distributed and clustered to high variance sections—which enables us to also consider small changes of energy demand. Note that this figure shows the raw data. In order to compute the entropy, we count the number of measurements in each interval and compute the probability by dividing through the total number of intervals. Next, we compute the entropy using the probabilities as input data.

We found that large intervals react well on changing variance and reliably show the start and end of each phase, while many small intervals may emphasize to much on small changes. The clustering approach might be difficult to implement in real world scenarios, since the clusters would differ for each household and the distribution of cluster centres may show legitimate changes each month.

Overall, our method assumes human activity to fall in a certain range of the entropy and, hence, defines a threshold to split the metric. The binary result r∈{0,1} depends on a threshold ε.

(6) $$r=\left\{\begin{array}{cc} 1 & \;\mathrm{if}\;H\;\mathrm{is}\;>\varepsilon \\ 0 & \;\mathrm{if}\;H\;\mathrm{is}\;\leq \varepsilon \end{array}\right.$$

Note that the interpretation of the sliding window entropy and interval entropy is oppositional and, hence, the meaning of *r* changes. If the sliding window entropy is higher ε, then the energy demand is equally distributed over many sliding windows and, hence, does not contain the peaks that indicate human activity. If the interval entropy is higher ε, then the measurements are distributed over all intervals and, hence, have a high variation, which indicates human activity.

In summary, the sliding window entropy depends upon the size and overlap of the window, while the interval entropy depends upon the number of intervals and distribution of intervals. After computing the probabilities with these parameters, both of the methods can be further tuned with the number of probabilities used as input. As the values in our metric are always relative to each other, less input values mean that an outlier that leads to a low entropy is usually more visible. The parameter tuning can be seen as an optimization problem. In the next section, we graphically analyse the influence of the parameters, but a detailed method to automatize this process for different households is beyond the scope of this paper.

## 5. Results

In the following experiments, we aim to detect occupancy while using real world measurements and compare our method with other well established prediction schemes. We first introduce our method to create a ground truth by labelling occupancy using appliance level data. Next, we predict these ground truth data without access to the appliance level data. Note that the ground truth data cannot be reliably created without the appliance level data, which are not available for the algorithms that aim to detect the occupancy.

We use the ECO dataset provided by Kleiminger et al. [5], which is a comprehensive dataset for non-intrusive load monitoring. The dataset offers individual appliance readings every second (Hz). The measurements used here are mean-cycle-power measurements of the real power (W), as provided by smart meters. The data are measured at the plug level for individual appliances and, apart from declared exceptions, provides measurements in Watt with four decimal places. Because we do not attempt to detect individual appliance signatures, we aggregated the values to one measurement every minute, which is due to the privacy concerns a more realistic interval for measurements in real world smart grids.

We used the appliance-level data of all six household for summer (2012 June to 2012 October) and winter (2012 October to 2013 January) in order to create the ground truth data. We labelled events that point to occupancy by selecting appliances with non-periodic behaviour (7c) and exclude appliances in stand-by (7a) mode, appliances running permanently and appliances with regular energy pattern (7b) (e.g., freezer, fridge, …).

Consider each of the *i* appliances in the ECO dataset as finite time series $T_{i}=\left\{a_{0}\ldots a_{n}\;|\;a\in R\right\}$, with *n* elements. We select each appliance $T_{i}$ that meets following conditions:

(7a) $$\begin{array}{cc} C_{1} & =max(T_{i})>10 \end{array}$$

(7b) $$\begin{array}{cc} C_{2} & =Q2\left(T_{i}\right)-min\left(T_{i}\right)<max\left(T_{i}\right)-Q2\left(T_{i}\right) \end{array}$$

(7c) $$\begin{array}{cc} C_{3} & =Q3\left(T_{i}\right)-Q2\left(T_{i}\right)<max\left(T_{i}\right)-Q3\left(T_{i}\right) \end{array}$$

Note that, *Q*2 denotes the second quantile, which is the median, and *Q*3 is the third quartile which is the middle value between the median and max. If an appliance meets these conditions, than all values greater than the arithmetic mean value of the appliance were labelled as occupancy.

We compare the entropy inspired metric with two methods provided by [16], namely the and (GeoMa).

With the data outlined above, we compare our own method to four well-known algorithms, namely (a) Page–Hinkley test, (b) Geometric Moving Average (c) Moving Average, (d) Seasonal Decomposition, and the (e) Standard Deviation. Each algorithm aims to classify occupancy (true or false) for 15 min time windows.

The first two algorithms are provided by Becker et al. [16], who performed occupancy detection on the same dataset. The method (a) Page–Hinkley Test (PHT) is a change detection algorithm, while (b) the Geometric Moving Average (GeoMA) detects any value higher than the average as occupied. Method (c) detects whether a measurement has a demand higher or lower than the Moving Average (MA), plus an adjustment value, (d) uses the trend component of Seasonal Decomposition (SD) [25] and detects values higher than a threshold. The last method, (e) detects whether the standard deviation is higher than the daily main. Our two original algorithms are evaluated, as follows, the sliding window entropy is using five minutes to compute a probability for the 15 min time window, whereas the interval entropy is using 20 uniformly distributed intervals. Note that we had to accept some limitations due to the different input and output data of each algorithm. Namely, some of the algorithms require a time window as input data, while others generate a decision for each input. For the latter, we aggregated the measurements to 15 min intervals and did not find a decreased performance, which is in consensus with Becker et al. [16]. Furthermore, we usually evaluate our algorithms with the so-called Area under the curve (AUC) of a receiver operating characteristic (ROC) curve in order to avoid to falsify results due to a non-optimal threshold. The curve shows the sensitivity (true positive rate) and the specificity (true negative rate) for any possible threshold.

The sensitivity is given by $\frac{TP}{TP+FN}$, while the specificity is given by $\frac{TN}{TN+FP}$. The accuracy of a method can be defined by the AUC. A good method can maximize both sensitivity and specificity, which results in a big area under the curve, while a random method results in a diagonal line and AUC of 0.5.

However, this is not possible for all algorithms as some of them require the threshold throughout the computation and only return a decision instead of an anomaly score. We still computed the AUC with a boolean result, (e.g., to perform a statistical test), but had to optimize the threshold for each function manually using linear optimization on the summer half of the first household of the ECO dataset.

In Table 1, we computed the AUC by evaluating the 96 results per day (15 min intervals) with the expected ground truth. A good method can maximize both sensitivity and specificity, which results in a big area under the curve, while a random method results in a diagonal line and an AUC of 0.5. Hence, the probability that an algorithm assigns the correct classification can be defined by the area under the curve (AUC). Table 1 shows the performance of each algorithm. The decomposition performed very good on this month, but we were not able to adjust the threshold for a good performance on the complete dataset. It might be possible to increase the performance by dynamically finding a new threshold after a fixed time period. However, this is not in the scope of this work.

**Figure 5:**
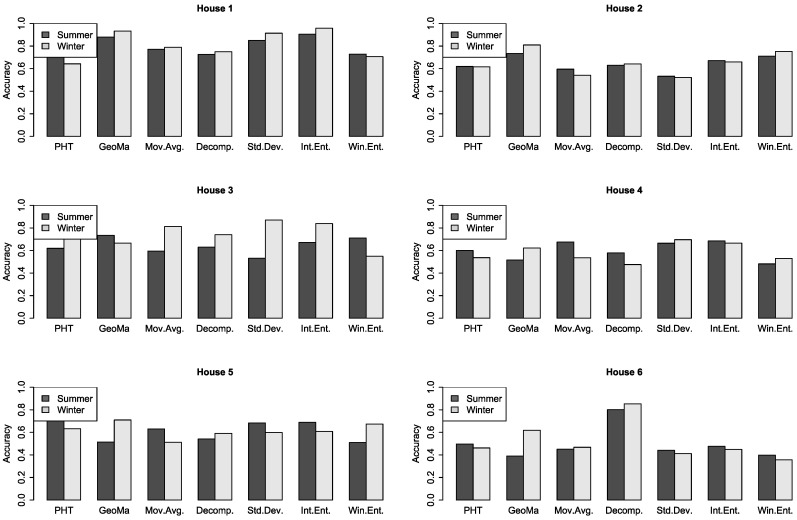
Accuracy for each household of the ECO dataset.

Figure 5 shows the results on a larger time scale and for all households. Each bar visualizes the result of an algorithm in summer and winter. The results are given in Accuracy (ACC), which is computed with a confusion matrix, given by (tp+tn)/(tp+tn+fp+fn), which returns the performance in percentage.

Next, we perform a pairwise bootstrap test (see Table 2) with the results of two methods, in order to evaluate the significance of the AUC over the summer in household 1. The bootstrap test re-samples the original results repeatedly (N=2000), which approximately follows a normal distribution, in order to perform a hypothesis test. Here, the null hypothesis is that both methods perform equally and the alternative hypothesis is that the AUC of both methods are different. A small *p*-value shows that the null hypothesis can be rejected.

Last but not least, we evaluated the parameters for the interval entropy and sliding window entropy. Here, we used the same input parameters as above to compute the AUC, but for different time windows and in the case of the interval entropy also for different numbers and types of intervals (note that, a time window of 60 Minutes means that we compute the entropy 24 times per day).

**Figure 6:**
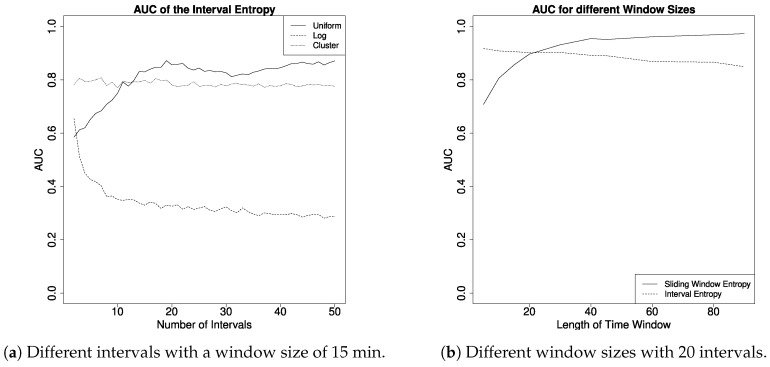
Effect of parameters on the interval entropy.

In Figure 6, we can see the influence of parameters on the AUC of the interval entropy in detail. The output varies depending on the number of intervals (left plot), where each line shows a different type to distribute the intervals. Here, the uniform distribution showed the best results and it is maximized with about 20 intervals and time windows of 30 min. Next, we compare the AUC of the interval entropy (20 uniform intervals) and sliding window entropy with different window sizes. The sliding window entropy performs better with larger time windows, while the AUC of the interval entropy is slowly decreasing with greater window sizes. The interval entropy works on time periods of arbitrary length. Although using it on long periods is possible (e.g., to detect absence on vacation or incoming tenants), we did not test the metric for such parameters. We think that it is more desirable to have an higher output resolution and repeat the process several times until the complete data is covered. When adjusting parameters, such as the length of input and output, for both the interval and sliding window entropy, it is intuitive to think of entropy as the probability that all input values are similar. If more probabilities are used as input values, we have more similar values and the entropy is generally higher. Hence, the difference between a normal and anomalous entropy is smaller, which makes it challenging to define a good threshold. The parameter tuning obviously depends on the actual household and slightly changes every time. However, we can clearly see that especially the sliding window entropy does not depend on fine granular data, as one measurement per time window is theoretically enough for this method to work.

## 6. Conclusions and Outlook

In this paper, we presented a non-intrusive approach to detect occupancy without a priori data. Our first results compared the feasibility of an entropy inspired metric while using different parameter. Our results showed a significantly reduced noise, outlier resilience and clearly visible usage patterns. Both of the methods reflect changes in energy demand with slightly different purpose. The sliding window entropy accurately shows on-/off behaviour and peaks of appliances while hiding less interesting absolute values. The interval entropy highlights high variance sections of a time-series while masking static behaviour. Both of the methods appear to behave well with a moderate number of input values. Our experiments utilizing the ECO dataset motivated the real world suitability.

In the graphical representation of the accuracy for all households, the results vary for all households as well as winter and summer. The differences for winter and summer were very different for each household, only ‘house 3’ performed clearly better during winter. Altogether, we received results varying from 0.7 to above 0.9. For ‘house 1’, which used the set of the parameters for all methods, we reached an accuracy higher 0.9. This is an indicator that future approaches can improve the results by tuning the parameter for a certain household, distinguish seasons, and perhaps distinguish weekend and weekdays. In our experiments, the PHT and SA also performed good and similar to the entropy look at the randomness of the energy demand. The interval entropy performed better then the sliding window entropy, but the parameter tuning was more difficult, and we experienced more noise in the performance depending on the type and number of intervals.

In the experimental evaluation, the entropy could not clearly exceed the results of all other methods. However, we had to accept many restrictions in order to compare our methods to other methods (e.g., the resolution, window sizes, and required boolean output) and think it is possible to further increase the performance by fine-tuning some parameters. Here, our focus was set on the entropy as a metric, which is intuitive to interpret and valuable as a numeric value to analyse and understand energy demand. We aimed to presented the fundamental knowledge necessary to apply this new metric to other applications of energy demand, such as energy theft detection and non-intrusive load monitoring.

We believe that the ’information content’ is profoundly affected by many energy related events and, hence, the entropy should be generally suitable for an an eclectic range of classification and identification tasks when combined with appropriate parameters. As future prospects, we would like combine our metric with tasks, such as non-intrusive load monitoring, anomaly detection, and energy demand forecasting. 

## Figures and Tables

**Table 1:** Results of all methods (AUC).

	PHT	GeoMa	MA	SA	Std. Dev.	Int. Ent.	Win. Ent.
2012-06-01	0.837	0.765	0.648	0.868	0.754	0.897	0.912
2012-06-02	0.511	0.744	0.559	0.67	0.534	0.506	0.744
2012-06-03	0.672	0.758	0.691	0.823	0.711	0.871	0.806
2012-06-04	0.457	0.815	0.672	0.967	0.955	0.803	0.809
2012-06-05	0.747	0.794	0.688	0.824	0.763	0.793	0.831
2012-06-06	0.723	0.875	0.823	0.913	0.779	0.898	0.905
2012-06-07	0.602	0.63	0.672	0.69	0.58	0.637	0.598
2012-06-08	0.845	0.802	0.746	0.893	0.81	0.942	0.885
2012-06-09	0.477	0.744	0.749	0.824	0.861	0.917	0.718
2012-06-10	0.853	0.67	0.612	0.896	0.641	0.702	0.79
2012-06-11	0.836	0.664	0.793	0.867	0.745	0.9	0.817
2012-06-12	0.966	0.881	0.581	0.874	0.784	0.897	0.924
2012-06-13	0.618	0.821	0.669	0.875	0.685	0.779	0.769
2012-06-14	0.707	0.692	0.668	0.832	0.784	0.879	0.778
2012-06-15	0.416	0.649	0.417	0.409	0.549	0.504	0.513
2012-06-16	0.8	0.908	0.748	0.922	0.829	0.95	0.975
2012-06-17	0.824	0.548	0.499	0.5	0.952	0.537	0.702
2012-06-18	0.831	0.789	0.682	0.923	0.717	0.856	0.885
2012-06-19	0.716	0.846	0.677	0.9	0.752	0.837	0.91
2012-06-20	0.699	0.793	0.622	0.896	0.637	0.692	0.919
2012-06-21	0.735	0.656	0.579	0.712	0.588	0.626	0.756
2012-06-22	0.592	0.626	0.523	0.532	0.516	0.51	0.479
2012-06-23	0.79	0.763	0.693	0.943	0.823	0.868	0.769
Total (Avg.)	0.707	0.749	0.653	0.807	0.728	0.774	0.791

**Table 2:** Bootstrap test for all methods (AUC).

$H_{1}$	AUC1	AUC2	*p*-Value
PHT vs. GeoMA	0.718	0.779	$5.28\times 10^{-26}$
PHT vs. MA	0.718	0.639	$8.83\times 10^{-46}$
PHT vs. SD	0.718	0.661	$3.28\times 10^{-21}$
PHT vs. Std. Dev.	0.718	0.722	$5.70\times 10^{-1}$
PHT vs. Int. Ent.	0.718	0.745	$2.15\times 10^{-5}$
PHT vs. Win. Ent.	0.718	0.714	$5.61\times 10^{-1}$
GeoMA vs. MA	0.779	0.639	$2.24\times 10^{-136}$
GeoMA vs. SD	0.779	0.661	$1.88\times 10^{-70}$
GeoMA vs. Std. Dev.	0.779	0.722	$2.84\times 10^{-20}$
GeoMA vs. Int. Ent.	0.779	0.745	$9.83\times 10^{-11}$
GeoMA vs. Win. Ent.	0.779	0.714	$3.60\times 10^{-29}$
MA vs. SD	0.639	0.661	$7.30\times 10^{-4}$
MA vs. Std. Dev.	0.639	0.722	$8.44\times 10^{-33}$
MA vs. Int. Ent.	0.639	0.745	$3.45\times 10^{-58}$
MA vs. Win. Ent.	0.639	0.714	$3.87\times 10^{-29}$
SD vs. Std. Dev.	0.661	0.722	$1.59\times 10^{-16}$
SD vs. Int. Ent.	0.661	0.745	$7.48\times 10^{-33}$
SD vs. Win. Ent.	0.661	0.714	$4.40\times 10^{-11}$
Std. Dev. vs. Int. Ent.	0.722	0.745	$1.12\times 10^{-8}$
Std. Dev. vs. Win. Ent.	0.722	0.714	$2.65\times 10^{-1}$
Int. Ent. vs. Win. Ent.	0.745	0.714	$4.88\times 10^{-6}$

## Abbreviations

The following abbreviations are used in this manuscript:

H	Entropy
σ	Standard Deviation (Std. Dev.)
Δ	Sliding Step
T	Time Series
X	Sliding Window
I	Interval
R	Real Numbers
Hz	Hertz (unit frequency)
W	Watt (unit power)
P	Probability
TP	True Positive
TN	True Negative
FP	False Positive
FN	False Negative
PHT	Page-Hinkley Test
AUC	Area under the curve
ROC	Receiver Operating Characteristic
ECO	Electricity Consumption & Occupancy
GeoMA	Geometric Moving Average
MA	Moving Average
SA	Seasonal Decomposition
